# Comparison of adaptation characteristics between visually and memory-guided saccades

**DOI:** 10.1152/jn.00050.2024

**Published:** 2024-06-12

**Authors:** Yoshiko Kojima, Hidetaka Yoshino, Leo Ling, James O. Phillips

**Affiliations:** ^1^Department of Otolaryngology-HNS, University of Washington, Seattle, Washington, United States; ^2^Department of Physiology and Biophysics, University of Washington, Seattle, Washington, United States; ^3^Washington National Primate Research Center, University of Washington, Seattle, Washington, United States; ^4^Virginia Merrill Bloedel Hearing Research Center, University of Washington, Seattle, Washington, United States

**Keywords:** adaptation, internally driven movement, monkey, saccade, visuospatial working memory

## Abstract

Saccade adaptation plays a crucial role in maintaining saccade accuracy. The behavioral characteristics and neural mechanisms of saccade adaptation for an externally cued movement, such as visually guided saccades (VGS), are well studied in nonhuman primates. In contrast, little is known about the saccade adaptation of an internally driven movement, such as memory-guided saccades (MGS), which are guided by visuospatial working memory. As the oculomotor plant changes because of growth, aging, or skeletomuscular problems, both types of saccades need to be adapted. Do both saccade types engage a common adaptation mechanism? In this study, we compared the characteristics of amplitude decrease adaptation in MGS with VGS in nonhuman primates. We found that the adaptation speed was faster for MGS than for VGS. Saccade duration changed during MGS adaptation, whereas saccade peak velocity changed during VGS adaptation. We also compared the adaptation field, that is, the gain change for saccade amplitudes other than the adapted. The gain change for MGS declines on both smaller and larger sides of adapted amplitude, more rapidly for larger than smaller amplitudes, whereas the decline in VGS was reversed. Thus, the differences between VGS and MGS adaptation characteristics support the previously suggested hypothesis that the adaptation mechanisms of VGS and MGS are distinct. Furthermore, the result suggests that the MGS adaptation site is a brain structure that influences saccade duration, whereas the VGS adaptation site influences saccade peak velocity. These results should be beneficial for future neurophysiological experiments.

**NEW & NOTEWORTHY** Plasticity helps to overcome persistent motor errors. Such motor plasticity or adaptation can be investigated with saccades. Thus far our knowledge is primarily about visually guided saccades, an externally cued movement, which we can make only when the object is visible at the time of saccade. However, as the world is complex, we can make saccades even when the object is not visible. Here, we investigate the adaptation of an internally driven movement: the memory-guided saccade.

## Graphical abstract

**Figure:**
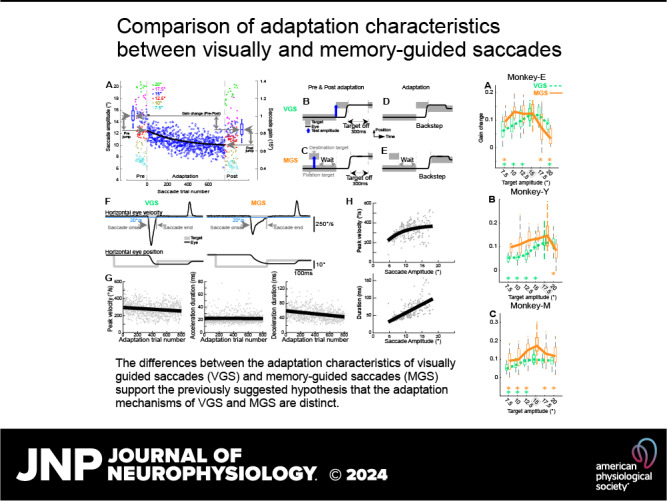

## INTRODUCTION

The object of interest is not always visible at the time of movement. Visuospatial working memory plays an essential role in directing movements in such situations. This system stores the position of an object in the visual world and can be used to direct a movement to that location. This type of movement is an internally driven movement. In contrast, we make another type of movement, an externally cued movement, when the object is visible at the time of movement. To make movements accurate in both situations, visuospatial working memory and the visual signal need to be appropriately transformed into a motor command signal.

Motor adaptation plays an essential role in maintaining motor accuracy. The behavioral characteristics and neural mechanisms of motor adaptation for an externally cued movement are well studied in nonhuman primates; however, little is known about motor adaptation for an internally driven movement. Saccades, rapid eye movements that direct the gaze to targets of interest (1), can be induced by an external visual signal (visually guided saccade, VGS) or by an internal signal related to visuospatial working memory (memory-guided saccade, MGS). Also, saccades are very precise. Because saccades remain accurate throughout life despite neural and muscular changes due to aging or injury (2–4), the saccadic system must be continually recalibrated through saccade adaptation. Previous studies suggest that the neural mechanism for MGS adaptation is distinct from that for VGS adaptation (5–7). First, when VGS were examined after MGS adaptation, VGS remained unchanged. Second, the VGS and MGS adaptation time courses are different. To gain insight into the MGS adaptation mechanism, here we compared the details of saccade dynamics during VGS and MGS adaptations in nonhuman primates. We employed nonhuman primates in preparation for future neurophysiological experiments. Furthermore, previous studies on VGS showed that the gain change was confined to a limited range of saccade amplitudes around the adapted target amplitude (a so-called “adaptation field”) (8, 9). In this study, we also examined whether or not an adaptation field with similar characteristics exists in MGS.

## MATERIALS AND METHODS

All experiments were performed in accordance with the *Guide for the Care and Use of Laboratory Animals* and exceeded the minimum requirements recommended by the Institute of Laboratory Animal Resources and the Association for Assessment and Accreditation of Laboratory Animal Care International. All procedures were evaluated and approved by the Institutional Animal Care and Use Committee of the University of Washington.

### Surgery and Training

Three *Macaca mulatta* monkeys (*monkeys E*, *M*, and *Y*) participated in this study. We implanted each monkey with fixtures to prevent head movements and a scleral search coil (10) to measure eye position.

After the monkeys recovered from the surgery, we trained them to track a small visual target in a dimly lit, sound-attenuating booth. The target was a 0.3° laser spot projected onto a tangent screen via two computer-controlled orthogonal mirror galvanometers. The screen was 65 cm from the monkey’s eyes. The monkey sat in a primate chair with its head restrained. We measured eye position with the electromagnetic search coil method (11). We rewarded the monkeys with fortified applesauce for keeping their gaze within ±2° windows around the horizontal and vertical positions of the target spot for at least 0.5 s. Once they had learned to fixate the target spot, we trained them to make visually guided saccades (VGS) to a stepping spot that moved to random locations on the tangent screen within a ±18° radius from the point on the screen straight ahead of the monkey. We also trained them to make memory-guided saccades (MGS). In this paradigm, the monkey first had to fixate a target for 0.3 s, and then we turned on a destination target briefly for 0.15 s; 1.0 or 1.2 s after the destination target was extinguished, the fixation target was also turned off, serving as a go signal for the monkey to make a saccade to the remembered location of the destination target. When the saccade landed at the remembered target location, the destination target turned on again. In this paradigm, the monkey needed to maintain memory of the destination target location for 1.0 or 1.2 s. We randomly mixed two wait durations (1.0 and 1.2 s) so the monkey could not predict the time of the go signal. For both types of saccades, we delivered the applesauce reward (∼0.16 mL per dollop, ∼200 mL/h) by a pump (Masterflex tubing pump, Cole-Parmer, Vernon Hills, IL) every 2 s regardless of the amplitude, direction, or timing of the saccade, as long as it landed within the ±2° window surrounding the target. The saccade was required to occur within 0.6 s of the go signal, and the subsequent fixation had to be maintained for at least 0.3 s for a reward to be given.

Finally, we trained VGS and MGS adaptations. For each monkey, we induced adaptations for 15° and 17.5° primary target steps to determine which amplitude induced greater adaptation in both saccade types: 15° was better in *monkeys E* and *M*, and 17.5° was better in *monkey Y*.

### Experimental Procedures

In an experiment, we examined saccade adaptation in one direction along the horizontal meridian (rightward or leftward) in one of two saccade types (VGS or MGS). The test saccade type was randomly selected. We performed one experiment per day and induced adaptation in one direction only. The primary saccade direction to be adapted was varied such that the same direction was never adapted in consecutive experiments. For the other direction, we did not induce adaptation, to eliminate any remaining effect of a previous adaptation (the so-called “washout” of the previous adaptation) (12, 13). For this washout direction, we randomly mixed two primary target step amplitudes (15° and 17.5°). All together, we collected 12 VGS and 12 MGS experiments in *monkey E*, 12 VGS and 12 MGS experiments in *monkey Y*, and 23 VGS and 22 MGS experiments in *monkey M*.

**Figure 1.**
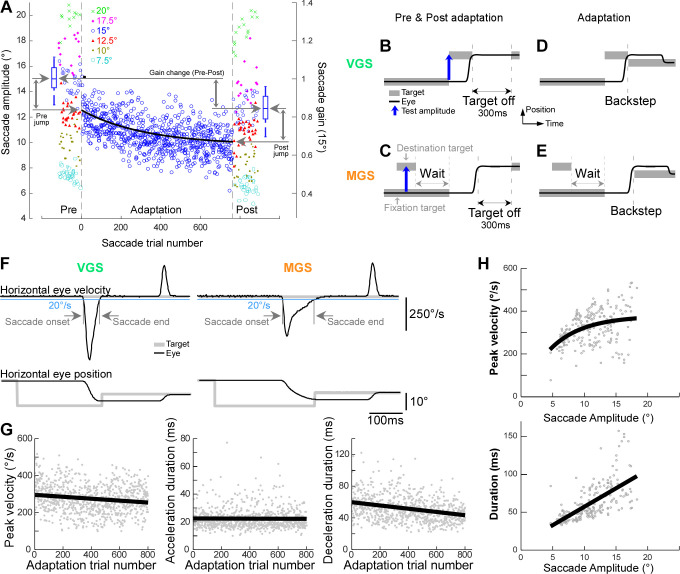
Adaptation paradigm. *A*: representative experiment of memory-guided saccade (MGS) adaptation (*monkey E*, rightward saccades): saccade amplitude during Preadaptation, Adaptation, and Postadaptation as a function of saccade trial number. Each dot represents an individual saccade; each color represents each primary target step size (7.5, 10, 12.5, 15, 17.5, 20°). Black exponential line is an exponential fit. *Right y*-axis indicates gain for saccades to 15° primary target steps. *Left* and *right* blue box plots indicate the median saccades gain during pre- and postadaptation, respectively, for 15° primary target steps. Gray vertical arrow with “Pre jump”: gain change at the beginning of adaptation session (= median of the box plot to the beginning of the exponential line). Gray vertical arrow with “Gain change”: gain change during adaptation session (= beginning to the end of the exponential line). Gray vertical arrow with “Post jump”: gain change at the beginning of postadaptation (= end of the exponential line to the median of the box plot). *B*: pre- and postadaptation paradigm for visually guided saccades (VGS). *C*: pre- and postadaptation paradigm for MGS. *D*: adaptation paradigm for VGS. *E*: adaptation paradigm for MGS. Gray thick horizontal line: target position; black solid line: eye position. Blue vertical arrow: primary target step amplitude, which was randomly varied (7.5, 10, 12.5, 15, 17.5, 20°) during pre- and postadaptation sessions. “Target off”: target was turned off for 300 ms after the saccade during pre- and postadaptation sessions. *F*: example traces for VGS and MGS to detect saccade onset and end time. *G*: representative experiment of saccade dynamics during MGS adaptation: saccade peak velocity (*left*), acceleration duration (*center*), and deceleration duration (*right*) as a function of saccade trial number. Each dot represents an individual saccade; black line is a linear regression fit. *H*: representative experiment of main sequence, MGS preadaptation: saccade peak velocity (*top*) and duration (*bottom*) as a function of saccade amplitude. Each dot represents an individual saccade; black line is an exponential (*top*) and a linear regression (*bottom*) fit.

Each experiment contains three blocks: a preadaptation block of ∼200 trials, an adaptation block of ∼800 trials, and a postadaptation block of ∼200 trials (Fig. 1*A*). During the pre- and postadaptation blocks, the monkey made saccades to target amplitudes of 7.5, 10, 12.5, 15, 17.5, or 20° (Fig. 1, *B* and *C*, blue vertical arrow, “Test amplitude”) in the test direction. At the end of each saccade, the target was turned off for 300 ms, so there was no postsaccadic visual error (Fig. 1, *B* and *C*, black horizontal arrow, “Target off”). In the adaptation block, we induced gain decrease adaptation of the saccade to a primary target step that empirically produced the greatest adaptation (15° in *monkeys E* and *M*, 17.5° in *monkey Y*) by providing a postsaccadic visual error (Fig. 1, *D* and *E*). We provided a constant visual error during the entire adaptation session by stepping the target 35% backward from the position where the saccade lands (7, 14, 15).

### Data Analysis

We digitized eye and target position signals at 1 kHz, using Power1401 data acquisition/controller hardware (Cambridge Electronic Design, Cambridge, UK). Data were saved to a hard drive for later analysis. A custom program running in Spike2 (Cambridge Electronic Design, Cambridge, UK) controlled target movement and the monkey’s reward via the Power1401 hardware.

A custom program running in Spike2 analyzed the saved data. It detected the occurrence of a saccade when eye velocity exceeded 75°/s within 70–800 ms after a go signal and marked saccade onset and end when the eye velocity exceeded or fell below 20°/s, respectively (Fig. 1*F*). The program measured saccade amplitude, peak velocity and duration, and the target distance before each saccade. The saccade and target attributes were exported to MATLAB (MathWorks, Natick, MA). Saccades whose initial eye positions differed from initial target positions by >5° were not analyzed, to eliminate trials in which animals were not fixating on the target at the beginning of the trial.

To analyze the changes in saccade amplitude during adaptation, we plotted it against the saccade trial number (e.g., Fig. 1*A*). To document the course of adaptation, we fitted the results with an exponential function (e.g., Fig. 1*A*, black exponential line) (5, 7, 16).

Adaptation course=a×exp (b×trial number)+c

To quantify gain change, we first determined the gain of each saccade:

Gain=(saccade amplitude/[target end position− eye start position])

Then, we calculated the median saccade gain to each primary target step size to obtain a gain change (Fig. 1*A*, “Gain change”):

Gain change=ηGainpre−ηGainpost

where ηGain is the median gain.

To quantify the “jump” at the beginning and end of the adaptation (Fig. 1*A*, “Pre jump” and “Post jump”), we calculated:

Pre jump=ηGainpre−Adaptation course (1)

$$\text{Post jump}=\eta {\text{Gain}}_{\text{post}}-\text{Adaptation course }\left(\text{end}\right)$$

where Adaptation course (1) is the exponential fit at trial number = 1. The adaptation course (end) is the value of the exponential fit at the last trial number during the adaptation block.

To compare the saccade latency (primary saccades and corrective saccades), we calculated the median latency across all adaptation trials.

To analyze the changes in saccade kinematics (peak velocity of saccade and duration of acceleration and deceleration phases) during adaptation, we plotted them against the saccade trial number (Fig. 1*G*). Acceleration duration is the time from saccade onset to the time of peak velocity. Deceleration duration is the time from peak velocity to the time of saccade end. To document the course of their changes, we fitted the results with a linear regression.

Saccade kinematics=a×trial number+b

To characterize the main sequence, we plotted the saccade peak velocity and duration against saccade amplitude and fitted them with an exponential function and linear regression, respectively (Fig. 1*H*) (16, 17).

Peak velocity=a×exp (b×amplitude)

Duration=a × amplitude+b

To display the adaptation field, that is, gain change transfer from the adapted amplitude to the other amplitudes, we plotted the median gain change against the primary target step size across experiments. The gain change of the adapted saccade was compared with the other five test amplitudes (Wilcoxon signed rank tests with Bonferroni correction for 5 times repetition, significant level: *P* < 0.05/5 = 0.01).

We also examined the adaptation field using percentage gain transfer.

Percentage gain transfer=Gain changetest target/Gain changeadapted target×100

To further characterize the adaptation field, we compared the adaptation field on either side of the adapted amplitude. For each experiment, we plotted the median gain change against the primary target step, separately on the left side for smaller amplitudes and on the right side for larger amplitude, and then fitted them with a linear regression.

Gain change=a×primary target step amplitude+b

## RESULTS

We induced gain decrease adaptation of visually guided saccades (VGS) and memory-guided saccades (MGS) and compared the adaptation characteristics in three monkeys.

**Figure 2.**
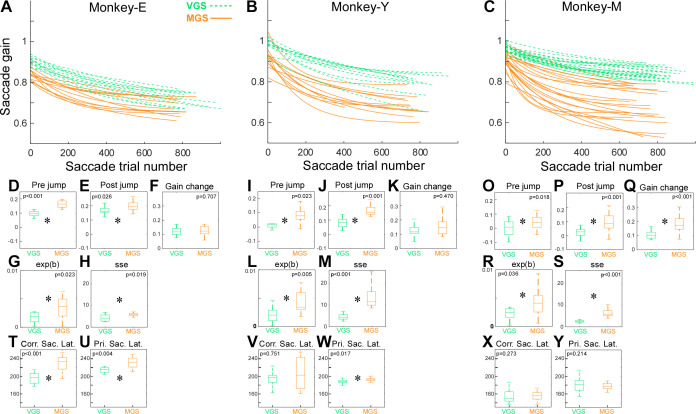
Adaptation time course. *A*: exponential fits on the relations of gain vs. saccade trial number during adaptation session for all experiments in *monkey E*. Orange: memory-guided saccades (MGS); green: visually guided saccades (VGS). *D–H*, *T*, and *U*: comparisons between VGS and MGS in Pre jump (*D*), Post jump (*E*), gain change (*F*), *b* of the exponential fit (rate constant = 1/*b*) (*G*), sum of squares error (sse) of the exponential fit (*H*), corrective saccade latency (*T*), and primary saccade latency (*U*) in *monkey E*. *P* values are from Wilcoxon rank-sum test. *B*, *I–M*, *V*, and *W*: *monkey Y*. *C*, *O–S*, *X*, and *Y*: *monkey M*. Same organization as in *A*, *D–H*, *T*, and *U*.

To determine the compatibility of this study with a previous study (12), we first compared the adaptation time courses between VGS and MGS. Figure 2, *A*–*C*, show the exponential fits to the relation between gain and the trial number during the adaptation session in all experiments for each monkey. In all three monkeys, the exponentials for MGS (orange solid lines) were below those for VGS (green dashed lines). This was because the MGS exhibited a sudden decrease at the beginning of the adaptation. This sudden decrease (“Pre jump”) in MGS was significantly greater across all monkeys (Fig. 2, *D*, *I*, and *O*; *P* < 0.001, *P* = 0.023, *P* = 0.018, respectively, Wilcoxon rank-sum test). The sudden increase at the beginning of the postadaptation (“Post jump”) was also significantly greater in MGS across all monkeys (Fig. 2, *E*, *J*, and *P*; *P* = 0.026, *P* <0.001, *P* <0.001, respectively, Wilcoxon rank-sum test).

The gain change in MGS and VGS were not significantly different in *monkeys E* and *Y*, but that of MGS was significantly greater than that of VGS in *monkey M* (Fig. 2, *F*, *K*, and *Q*; *P* = 0.707, *P* = 0.470, *P* < 0.001, respectively, Wilcoxon rank-sum test).

The curvature of the exponential line was sharper in MGS than in VGS (Fig. 2, *A*–*C*). Indeed, the variable *b* of the exponential function (rate constant = 1/*b*) was significantly greater in MGS across all monkeys (Fig. 2, *G*, *L*, and *R*; *P* = 0.023, 0.005, 0.036, respectively, Wilcoxon rank-sum test). Another significant difference between MGS and VGS was the sum of squares error (sse) of the exponential fit. Across all monkeys, it was greater for MGS than for VGS (Fig. 2, *H*, *M*, and *S*; *P* = 0.019, *P* < 0.001, *P* < 0.001, respectively, Wilcoxon rank-sum test).

Finally, Fig. 2, *T–Y*, compare saccade latency between MGS and VGS. The corrective saccade latency in MGS and VGS were not significantly different in *monkeys Y* and M, but that of MGS was significantly longer than VGS in *monkey E* (Fig. 2, *T*, *V*, and *X*; *P* < 0.001, *P* = 0.751, *P* = 0.273, respectively, Wilcoxon rank-sum test). The primary saccade latency in MGS and VGS were not significantly different in *monkey M*, but that of MGS was significantly longer than VGS in *monkeys E* and *Y* (Fig. 2, *U*, *W*, and *Y*; *P* = 0.004, 0.017, 0.214, respectively, Wilcoxon rank-sum test).

In summary, all monkeys exhibited a greater jump at the beginning and end of the adaptation, a shorter rate constant, and a greater trial-by-trial variability in the MGS adaptation than in the VGS adaptation. These results were consistent with the previous study (12). In addition, there was an idiosyncratic difference in the amount of adaptation and saccade latency between monkeys.

**Figure 3.**
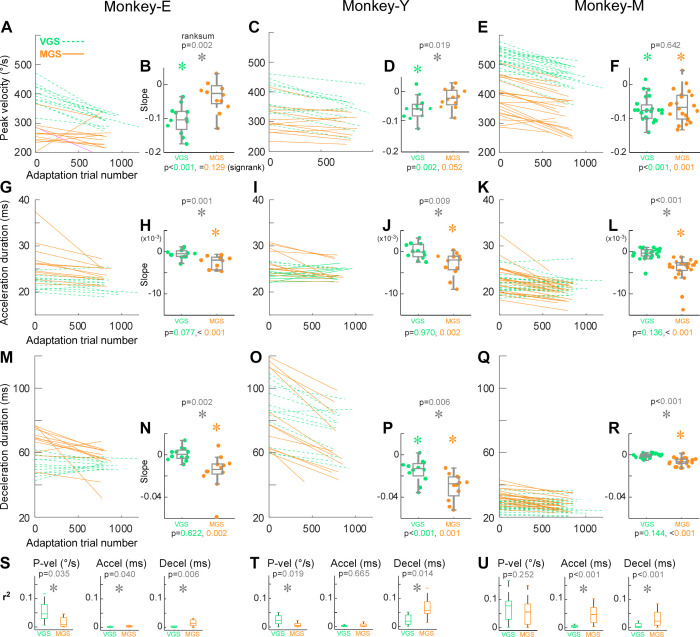
Change in the saccade dynamics during adaptation. *A*, *C*, and *E*: linear regression fits on the relation of peak velocity vs. saccade trial number during adaptation session for all experiments for each monkey. *B*, *D*, and *F*: the slopes of the regression line for all experiments: box plot (gray) and swarm plot (color dot). The gray *P* values at *top* and colored *P* values at *bottom* indicate the *P* value of Wilcoxon rank-sum test and Wilcoxon signed rank test, respectively. *Significant. *G–L*: acceleration duration. *M–R*: deceleration duration. Same organization as in *A–F*. *S*: *r*^2^ of the regression lines in *A* (peak velocity, *left*), in *G* (acceleration duration, *center*), and in *M* (deceleration duration, *right*). *T* and *U*: *monkeys Y* and *M*, respectively. MGS, memory-guided saccades; VGS, visually guided saccades.

Next, we examined the change in saccade dynamics during adaptation, that is, peak velocity and duration of acceleration and deceleration phases. Figure 3, *A–F*, compare the peak velocity between VGS and MGS. Figure 3, *A*, *C*, and *E*, show the fitted linear regression lines between peak velocity and trial number during all adaptation sessions for each monkey. The lines for MGS were below those for VGS, indicating that the peak velocity was lower during MGS than during VGS. To quantify the change during adaptation, we examined the slope of the regression lines. Across all monkeys, the slope of the VGS was significantly negative (Fig. 3, *B*, *D*, and *F*; *P* < 0.001, *P* = 0.002, *P* < 0.001, respectively, Wilcoxon signed rank test), suggesting that the peak velocity decreased during VGS adaptation. In contrast, the slope of MGS was not significantly different from 0 in *monkeys E* and *Y* but significantly negative in *monkey M* (Fig. 3, *B*, *D*, and *F*; *P* = 0.129, *P* = 0.052, *P* < 0.001, respectively, Wilcoxon signed rank test). The negative value of the slope was significantly smaller in MGS than in VGS in *monkeys E* and *Y*, but those of MGS and VGS were not significantly different in *monkey M* (Fig. 3, *B*, *D*, and *F*, *P* = 0.002, 0.019, 0.642, respectively, Wilcoxon rank-sum test).

Figure 3, *G–L*, compare the acceleration duration between VGS and MGS. Across all monkeys, the regression slope during VGS was not significantly different from 0 (Fig. 3, *H*, *J*, and *L*; *P* = 0.077, 0.970, 0.136, respectively, Wilcoxon signed rank test). In contrast, the slope during MGS was significantly negative in all monkeys (Fig. 3, *H*, *J*, and *L*; *P* < 0.001, *P* = 0.002, *P* < 0.001, respectively, Wilcoxon signed rank test). The negative value of the slope was significantly larger in MGS than in VGS across all monkeys (Fig. 3, *H*, *J*, and *L*; *P* = 0.001, *P* = 0.009, *P* < 0.001, respectively, Wilcoxon rank-sum test).

The deceleration duration did not change during VGS adaptation in *monkeys E* and *M* but decreased in *monkey Y*; in contrast, it decreased during MGS adaptation in all monkeys (Fig. 3, *M–R*). The negative value of the slope was significantly larger in MGS than in VGS across all monkeys (Fig. 3, *N*, *P*, and *R*).

Figure 3, *S–U*, show *r*^2^ value of the regression lines to compare the goodness of fit. The peak velocity fit was better in VGS, and those for acceleration and deceleration durations were better in MGS.

In summary, all monkeys exhibited a decrease in peak velocity during VGS adaptation and a decrease in acceleration and deceleration durations during MGS adaptation.

**Figure 4.**
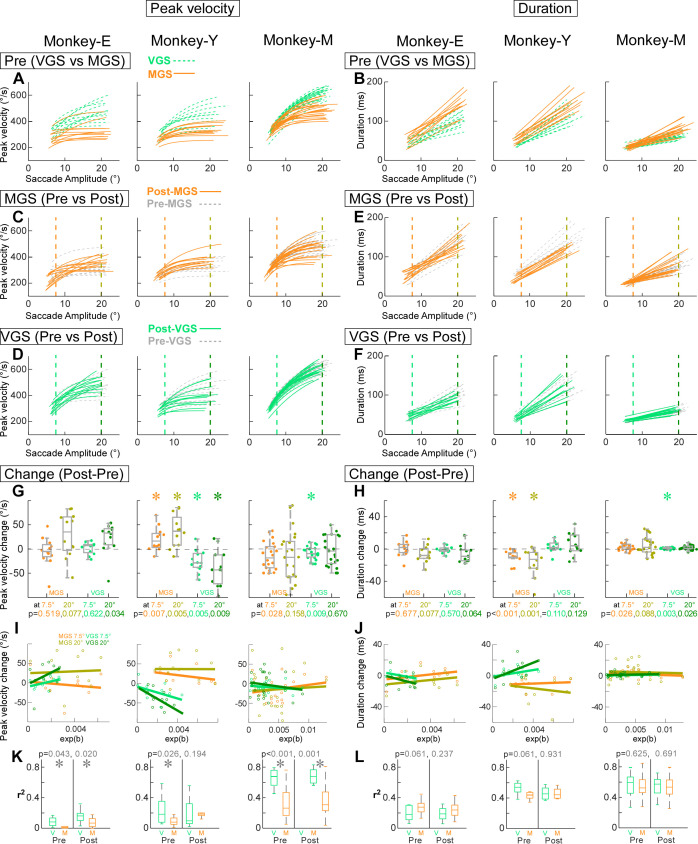
Change in the main sequence. *A*, *C*, and *D*: exponential fits on the relations of peak velocity vs. saccade amplitude for all experiments for each monkey (*left*: *monkey E*, *center*: *monkey Y*, *right*: *monkey M*) during preadaptation sessions to compare visually guided saccades (VGS) and memory-guided saccades (MGS) (*A*), and for MGS (*C*) and VGS (*D*) to compare pre- and postadaptation sessions. Two vertical dashed lines in each panel: saccade amplitude at 7.5° (*left*) and 20° (*right*). *B*, *E*, and *F*: linear regression fit on the relation of saccade duration vs. saccade amplitude for all experiments for each monkey. Same organization as in *A*, *C*, and *D*. *G*: the peak velocity change at 7.5° and 20°: box plot (gray) and swarm plot (color dot). The colored *P* values at *bottom* are from Wilcoxon signed rank test. *Significant. *H*: the saccade duration change at 7.5° and 20°. *I*: the peak velocity change (in *G*) as a function of *b* of the exponential fit (in Fig. 2, *G*, *L*, and *R*). *J*: the saccade duration change (in *H*) as a function of *b* of the exponential fit (in Fig. 2, *G*, *L*, and *R*). *K*: *r*^2^ of the exponential fits in *A*, *C*, and *D* (peak velocity) for *monkey E* (*left*), *monkey Y* (*center*), and *monkey M* (*right*). The gray *P* values at top are from Wilcoxon rank-sum test. *L*: *r*^2^ of the linear regression lines in *B*, *E*, and *F* (duration) for all monkeys.

We also compared the main sequence, that is, the relationship between saccade peak velocity and duration against saccade amplitude, before and after the VGS and MGS adaptations. Figure 4*A* shows the exponential lines fitted to preadaptation peak velocity. The lines for MGS were below those for VGS, indicating that the peak velocity was slower in MGS than in VGS. Figure 4*B* shows the regression lines fitted to saccade duration against saccade amplitude. The lines for MGS were above those for VGS, indicating that the durations were longer in MGS than in VGS.

Figure 4, *C–F*, compare the relationships pre- versus postadaptation. The lines pre- and postadaptation mostly overlap both for peak velocity (Fig. 4, *C* and *D*) and duration (Fig. 4, *E* and *F*). To examine these main sequence lines statistically, we compared the pre and post values exhibited at 7.5° and 20°. Figure 4*G* plots the difference (Post − Pre) of the values in peak velocity. At 7.5° in MGS for *monkey E* (Fig. 4*G*, *left*, leftmost column), the difference was −4.8 ± 25.0 [median and interquartile range (IQR)] and pre and post values were not significantly different (*P* = 0.519, Wilcoxon signed rank tests with Bonferroni correction of 4 times repetition: significant *P* < 0.05/4 = 0.0125). At 20° in MGS for *monkey E* (Fig. 4*G*, *left*, 2nd column on left), the peak velocity increased after the adaptation, by 35.7 ± 69.2 (median and IQR), but the difference was not significantly different (*P* = 0.077, Wilcoxon signed rank tests with Bonferroni correction of 4 times repetition). Similarly, there were no significant changes in VGS at 7.5° and 20° in *monkey E* (Fig. 4*G*, *left*, 2 right columns) (*P* = 0.622 and 0.034, Wilcoxon signed rank tests with Bonferroni correction of 4 times repetition). The main sequence changes in the duration were also not significant in *monkey E* (Fig. 4*H*, *left*). Thus, *monkey E* did not show any changes in the main sequence. For *monkey Y*, however, the peak velocity significantly increased at both 7.5° and 20° in the MGS and decreased at both amplitudes in the VGS (Fig. 4*G*, *center*). The duration exhibited the reverse change, that is, the duration decreased at both 7.5° and 20° in the MGS and increased at both amplitudes in the VGS (Fig. 4*H*, *center*). In *monkey M*, the peak velocity significantly decreased in the VGS at 7.5° (Fig. 4*G*, *right*) and the duration significantly increased in the VGS at 7.5° (Fig. 4*H*, *right*). In addition, we examined whether these changes are correlated to the adaptation rate (the variable *b* of the exponential function in Fig. 2, *G*, *L*, and *R*), but no consistent correlation was found (Fig. 4, *I* and *J*). Finally, Fig. 4, *K* and *L*, show the *r*^2^ values of the fitted lines to compare their goodness of fit. The peak velocity was better in VGS, but the duration was not different. In summary, the changes in the main sequence were different between monkeys; however, there were opposite patterns in the changes in peak velocity and duration.

**Figure 5.**
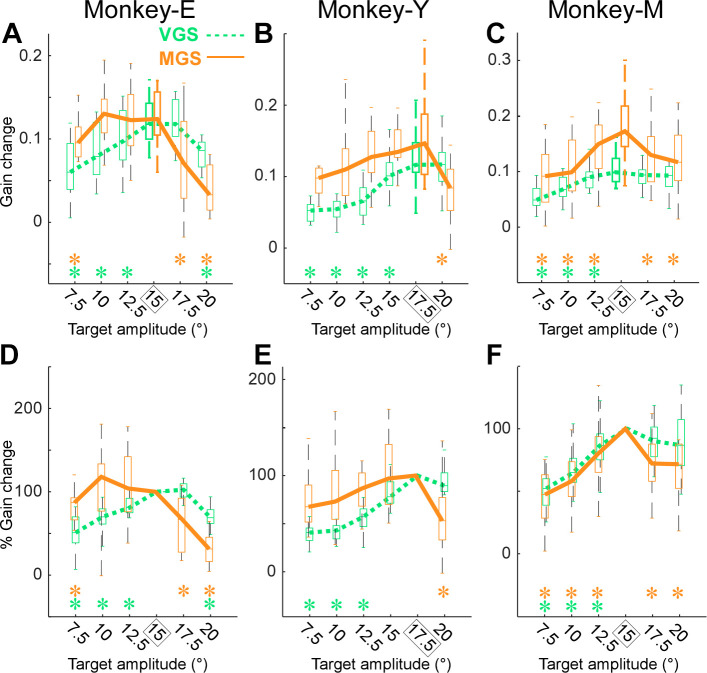
Adaptation field. *A*: gain change of saccade to 7.5, 10, 12.5, 15, 17.5, 20° horizontal primary target steps in *monkey E*; saccades to 15° primary target step were adapted. *Significant gain change (Wilcoxon signed rank test). *B*: *monkey Y*; saccades to 17.5° primary target step were adapted. *C*: *monkey M*; saccades to 15° primary target step were adapted. *D–F*: percentage gain transfer for each monkey. Same organization as in *A–C*. MGS, memory-guided saccades; VGS, visually guided saccades.

Finally, we examined the adaptation field, that is, the gain change transfer from the adapted saccade amplitude to other amplitudes. Figure 5*A* shows the VGS and MGS adaptation fields in *monkey E*. In VGS adaptation, the gain changes of all smaller amplitudes (7.5°, 10°, and 12.5°) and the largest amplitude (20°) were significantly smaller than that of the adapted saccade amplitude (15°) (*P* < 0.01, Wilcoxon signed rank tests with 5 times repeated Bonferroni correction: significant *P* < 0.05/5 = 0.01) (green asterisk in Fig. 5*A*). The gain change of one larger amplitude (17.5°) was not significantly different from that of the adapted saccade (15°) (*P* > 0.01, Wilcoxon signed rank tests with Bonferroni correction). Thus, consistent with the previous study (8, 9), VGS adaptation transferred less to the smaller amplitudes than to the larger amplitudes. In contrast, the adaptation field of MGS exhibited a reverse trend. The gain changes of the smaller amplitudes (10° and 12.5°) were not significantly smaller than that of the adapted saccade (15°) (*P* > 0.01, Wilcoxon signed rank tests with Bonferroni correction), but those of the smallest amplitude (7.5°) and both of the larger amplitudes (17.5° and 20°) were significantly smaller (*P* < 0.01, Wilcoxon signed rank tests with Bonferroni correction). *Monkey Y* also exhibited similar adaptation fields, that is, VGS adaptation transferred less to the smaller amplitude side and MGS adaptation transferred less to the larger amplitude side (Fig. 5*B*). *Monkey M* exhibited a VGS adaptation field similar to the other monkeys; however, MGS adaptation showed less transfer to both the smaller and larger sides (Fig. 5*C*).

Figure 5, *D–F*, show the adaptation field in terms of percentage gain transfer (see materials and methods). The results were the same.

**Figure 6.**
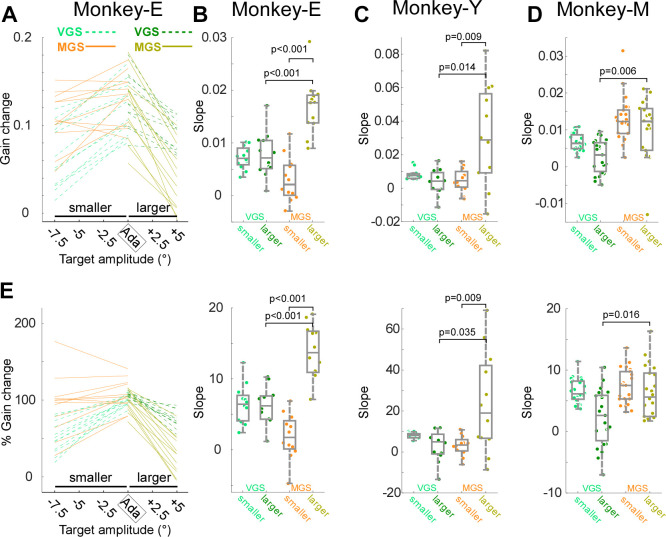
Adaptation transfer to the smaller and the larger sides. *A*: linear regression fits on the relation of gain change vs. target amplitude for all experiments in *monkey E*. Light green dashed and orange solid lines are the regression lines fitted on the adapted amplitude and smaller for visually guided saccades (VGS) and memory-guided saccades (MGS), respectively. Dark green dashed and dark yellow solid lines are the regression lines fitted on the adapted amplitude and larger for VGS and MGS, respectively. *B*: the slopes of the regression line for all experiments in *monkey E*: box plot (gray) and swarm plot (color dot). *P* values are the significant *P* values of Wilcoxon signed rank test (nonsignificant *P* values are not indicated). *C* and *D*: *monkeys Y* and *M*, respectively. Same organization as in *B*. *E*: percentage gain transfer. Same organization as in *A–D*.

To determine the difference in VGS and MGS on each side, we compared the linear regression lines fitted to the gain change for smaller target amplitudes and larger target amplitudes separately. Figure 6*A* shows the data of each experiment in *monkey E*. On the smaller side, most lines of the VGS (light green dashed) exhibited a positive slope; in contrast, only about half of MGS (orange solid lines) exhibited a positive slope. On the larger side, lines of the VGS (dark green dashed) and MGS (dark yellow solid) exhibited a negative slope, but the slopes were steeper in the MGS than in the VGS. For a statistical test, we plotted the slope values in Fig. 6*B*. To compare the values on both sides, we inverted the slope for the larger side, so the negative slopes are plotted as positive slopes. The inverted slope of the MGS adaptation on the larger side was significantly larger than the slope on the smaller side (*P* = 4.88 × 10^−4^, Wilcoxon signed rank test) and that of VGS adaptation on the larger side (*P* = 4.78 × 10^−4^, Wilcoxon rank-sum test). *Monkey Y* exhibited the same pattern of difference. The inverted slope of the MGS adaptation on the larger side was significantly larger than that on the smaller side (*P* = 0.0093, Wilcoxon signed rank test) and that of VGS adaptation on the larger side (*P* = 0.0141, Wilcoxon rank-sum test) (Fig. 6*C*). In *monkey M*, the inverted slope of the MGS adaptation on the larger side was not significantly larger than that on the smaller side, but it was significantly larger than that of VGS adaptation on the larger side (*P* = 0.0062, Wilcoxon rank-sum test) (Fig. 6*D*). Furthermore, the analysis with percentage gain transfer also showed the same difference (Fig. 6*E*). In summary, all monkeys exhibited a steeper decline in the MGS than in the VGS in the larger amplitudes. In the smaller side, there was no significant difference in the MGS and VGS.

## DISCUSSION

The objective of this study was to characterize MGS adaptation by comparing it with VGS adaptation. First, we showed differences in the adaptation time course, which was consistent with the previous study (12). Next, we found differences in the change of the saccade dynamics during adaptation. Finally, we characterized the differences in the adaptation field.

The adaptation time courses in this study were consistent with those reported in the previous studies. First, MGS adaptation exhibited a greater jump at the beginning and end of adaptation and a shorter time constant than VGS adaptation (Fig. 2) (7). As discussed in the previous studies, saccade adaptation consists of both a rapid and a slow process (12, 18, 19). MGS adaptation might involve the rapid process more than the slow process compared to the VGS adaptation. Second, the sum of squares error (sse) of the exponential fit was higher in MGS adaptation than VGS (Fig. 2) (7), because the MGS had a greater trial-by-trial variability (20).

The analyses of the saccade dynamics revealed a distinct difference in MGS and VGS adaptation (Fig. 3). During VGS adaptation, the peak velocity decreased as the saccade amplitude decreased, which is consistent with previous studies (21–23). In contrast, during MGS adaptation, the duration of acceleration and deceleration decreased as the saccade amplitude decreased. These results suggest that VGS adaptation uses a mechanism that decreases the peak velocity to decrease the saccade amplitude and MGS adaptation uses another mechanism that decreases the saccade duration to decrease the saccade amplitude. Note that previous studies showed that amplitude increase adaptation in VGS is accompanied by an increase in saccade duration (21–23), suggesting that the amplitude decrease and increase VGS adaptations are different. Thus, amplitude increase adaptation in MGS might also have different characteristics.

It is well known that VGS peak velocity has a distinct correlation with VGS amplitude, the so-called main sequence (1). To our knowledge, there is no previous study that has examined the change in the main sequence in MGS adaptation in monkeys. We found that each monkey showed idiosyncratic changes in main sequence in both VGS and MGS (Fig. 4).

The VGS adaptation field of this study (Fig. 5, Fig. 6) was consistent with previous studies (8, 9, 24). First, the greatest gain change occurred for the saccades whose amplitudes were nearest to those of the adapted saccades. Second, the gain change fell sharply for saccades that were smaller than the adapted saccades but more gradually for saccades that were larger in Fig. 5. One concern is that there are more primary target step amplitudes on the smaller side than on the larger side in this study. This might give the appearance of a sharper fall on the smaller side than on the larger side. However, a similar plot in the previous study (9), which examined the same number of primary target step amplitudes on both sides, indicated a similar result. Therefore, this concern is less likely to explain our results. Another concern is that the statistical test of Fig. 6 failed to detect the sharper fall on the smaller side. Perhaps this is because the sharpness of the fall is highly variable across experiments. Such variability has also been shown in the previous study (9).

This study demonstrated that although MGS adaptation also exhibits an adaptation field it exhibits characteristics different from VGS adaptation. Although the greatest gain change in MGS still occurred for saccades whose amplitudes were nearest to those of the adapted saccades, which is similar to the VGS, the gain change falls rapidly for saccades that were larger than the adapted saccades but more gradually for smaller saccades. These results suggest that the VGS adaptation transfers less to smaller amplitudes but the MGS adaptation transfers less to larger amplitudes.

### Speculation of MGS Adaptation Site

All results of this study and a previous study (7) showed that the adaptation characteristics of MGS are different from those of VGS, suggesting that the MGS adaptation site is different from that of VGS. Because the generation of VGS and MGS is thought to involve different brain regions (5, 25–28), it seems logical to expect that the VGS and MGS adaptation sites may also be distributed along the differing pathways of each saccade signal. Various studies have indicated that the VGS adaptation site is the oculomotor vermis (OMV) (29–35). Therefore, we speculate that the MGS adaptation is subserved one or more regions outside of the OMV, perhaps in addition to OMV.

Furthermore, because the MGS adaptation affects the VGS only partially, if at all (7), the MGS adaptation site(s) should be distributed primarily outside of the VGS signal route. Because the OMV exhibits the same activity pattern for VGS and MGS (36), the OMV should be involved in the VGS and MGS signal routes. This evidence further suggests that the MGS adaptation site is distributed primarily outside of the OMV. The superior colliculus (SC) exhibits a different firing pattern for MGS and VGS (37, 38). Upstream of the SC, structures such as basal ganglia, frontal eye field (FEF), supplementary eye field (SEF), dorsolateral prefrontal cortex (dlPFC), and lateral intraparietal area (LIP) have been implicated in MGS (38–44). The activity of LIP neurons does not change during MGS adaptation (44), suggesting that the MGS adaptation site is not in LIP. Thus, these observations together suggest that the MGS adaptation site(s) might be distributed across SC, basal ganglia, FEF, SEF, and/or dlPFC.

The saccade dynamics analysis suggested that MGS adaptation influences the saccade durations more than the peak velocity. The neurons in the substantia nigra pars reticulata (SNr), an output nucleus of the basal ganglia, pause their tonic activity for saccades (45), which might be able to control the saccade duration. However, the pause duration is not tightly correlated to saccade duration. Similarly, the duration of the saccade-related burst of the neurons in the SC and the cortical regions is not tightly correlated with the saccade duration. Therefore, these neurons are unlikely to be the top candidates. The activation of fixation neurons in the SC and FEF interrupts the ongoing saccades (46, 47) and their pause end correlated with the saccade end (48, 49). There are also fixation neurons in the dlPFC (50). Because these neurons might control the saccade duration and, consequently, decrease MGS gain during adaptation, it might be worth considering these neurons as candidates for future neurophysiological experiments.

### Data Variability

To control day-to-day variability, we repeated the experiment in each monkey. We did not interleave MGS and VGS adaptations in a single experiment to control for day-to-day variability because such a procedure would make the result uninterpretable, because VGS adaptation affects MGS (5–7). Testing MGS in the course of VGS adaptations would convolve changes due to both adaptive processes, not provide adaptation characteristics unique to MGS.

To examine differences between monkeys in adaptation, we collected both MGS and VGS data for each monkey. The main sequence analysis revealed that MGS adaptation was more idiosyncratic than VGS adaptation. This makes sense because MGS is considered a higher-order movement than VGS and involves more cortical activity (5). Furthermore, one monkey (*monkey M*) exhibited greater gain change in MGS adaptation trials than in VGS adaptation trials (Fig. 2). This monkey decreased the peak velocity and saccade duration during MGS adaptation, whereas the other two monkeys decreased only the saccade duration (Fig. 3). It is possible that this monkey had greater adaptation due to using both adaptation mechanisms during MGS adaptation: one to decrease the peak velocity and the other to decrease the duration.

### Comparing MGS and VGS

Conceptually, VGS involves two steps: visual signal processing and generation of the saccade command. It therefore involves only one transformation, visual to saccade, and the adaptation modulates the gain of this transformation. In contrast, MGS involves three steps: visual signal processing, visuospatial working memory, and generation of the saccade command. Therefore, two transformations are needed: visual to visuospatial working memory and visuospatial working memory to the saccade command. Because the LIP activity does not change during MGS adaptation (44), it is more likely that the later transformation is modulated by the MGS adaptation.

The involvement of visuospatial working memory is not the sole difference between VGS and MGS tasks. For example, the MGS task also must stop the saccade until a “go” signal occurs. We believe that this step does not influence the adaptation. For another type of saccade, the so-called “delayed saccade,” the subject also must hesitate after a visual target appears before moving to it. However, because the target never disappears, spatial memory is not required to make the delayed saccades. Current pieces of evidence suggest that VGS adaptation and delayed saccade adaptation employ the same adaptation mechanism. First, similar to VGS, the saccade-related bursts of the superior colliculus neurons exhibit no apparent change during the adaptation of delayed saccades (51, 52). Second, adaptation of VGS affects the gain of delayed saccades, and adaptation of delayed saccades affects VGS (7). Third, adaptations of either VGS or delayed saccades affect express saccades and scanning saccades similarly (7). These results suggest that they share a common adaptation mechanism, and halting the saccade does not affect the VGS adaptation process. Because VGS adaptation is the most studied saccade type, we think it is more reasonable to use VGS than delayed saccade to compare with MGS.

Finally, we used MGS as a model of internally driven movements. However, MGS is not the only internally driven movement; for example, antisaccades and scanning saccades can also be considered to be internally driven saccades. Each saccade type may use a different adaptation mechanism (7); therefore, the results of this study should not be interpreted to determine the mechanism of other internally driven saccade adaptation.

## DATA AVAILABILITY

Data will be made available upon reasonable request.

## GRANTS

This study was supported by National Institute of Health (NIH) Grants EY023277 and EY033760 (R01 for Y.K.). This work was also made possible by NIH Grants OD010425 (P51 for Washington National Primate Research Center) and P30EY001730 (Vision Research Core for University of Washington).

## DISCLOSURES

No conflicts of interest, financial or otherwise, are declared by the authors.

## AUTHOR CONTRIBUTIONS

Y.K. conceived and designed research; Y.K. and H.Y. performed experiments; Y.K. analyzed data; Y.K. interpreted results of experiments; Y.K. prepared figures; Y.K. drafted manuscript; Y.K., H.Y., L.L., and J.O.P. edited and revised manuscript; Y.K. approved final version of manuscript.
